# Hyperphosphatemia in an 11-year-old girl with acute myeloid leukemia: Questions

**DOI:** 10.1007/s00467-018-4097-x

**Published:** 2018-10-05

**Authors:** Monique Albersen, Arend Bökenkamp, Hans Schotman, Stephanie Smetsers

**Affiliations:** 1 Amsterdam UMC, Department of Clinical Chemistry, Vrije Universiteit Amsterdam, De Boelelaan 1117, 1081 HV Amsterdam, The Netherlands; 2 Amsterdam UMC, Department of Pediatric Nephrology, Vrije Universiteit Amsterdam, De Boelelaan 1117, 1081 HV Amsterdam, The Netherlands; 3 Amsterdam UMC, Department of Pediatric Oncology, Vrije Universiteit Amsterdam, De Boelelaan 1117, 1081 HV Amsterdam, The Netherlands; 4 Prinses Máxima Centrum voor Kinderoncologie, Heidelberglaan 25, 3584 CS Utrecht, The Netherlands

## Case presentation

An 11-year-old girl, recently diagnosed with acute myeloid leukemia, was treated according to the NOPHO DBH AML 2012 protocol [1]. After the third chemotherapy course, consisting of cytarabine, mitoxantrone, and intrathecal methotrexate, she was admitted to the Department of Pediatric Oncology because of septic shock during febrile neutropenia. She was treated with meropenem and vancomycin and blood cultures were positive for Streptococcus mitis. Because of persistent fever, the central venous catheter was removed. Nevertheless, the fever persisted and a chest CT was performed, which revealed multiple abnormalities suggestive of pulmonary aspergillosis, which was confirmed by bronchoalveolar lavage (BAL). On day 5 of admission, she was started on AmBisome® (liposomal amphotericin B; 5 mg/kg in glucose 5%). Because of her persisting neutropenia, granulocyte colony-stimulating factor (G-CSF) was administered. Fever disappeared with neutrophil recovery, approximately 5 days after the start of G-CSF and AmBisome®.

**Fig. 1:**
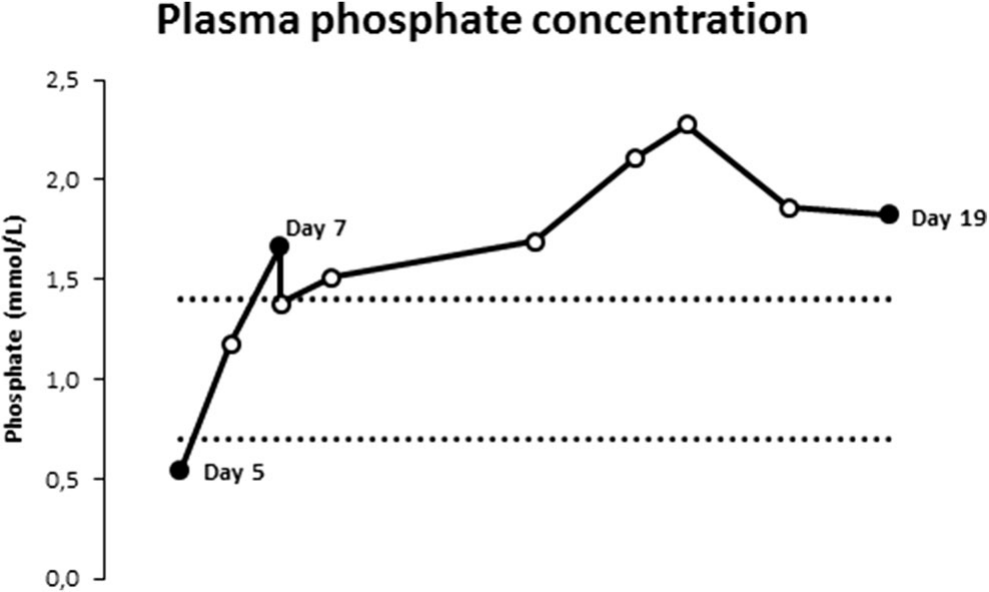
Course of phosphate concentrations. The dotted lines represent the reference range (0.70-1.40 mmol/L)

Repeated blood tests showed normal renal function (creatinine 35 μmol/L, urea 3.7 mmol/L). Potassium supplementation was started because of hypokalemia. While phosphate concentrations were low at 0.54 mmol/L on day 5, they rose spontaneously and from day 7 onwards laboratory tests showed progressive hyperphosphatemia, with a maximum of 2.28 mmol/L (Fig. 1).

## Question

What is the most likely cause of the hyperphosphatemia observed in this patient?

## References

[1] NOPHO-DBH AML (2012) Protocol: research study for treatment of children and adolescents with acute myeloid leukaemia 0-18 years. EudraCT Number 2012-002934-35 (https://www.clinicaltrialsregister.eu/ctr-search/search?query=nopho+dbh+aml)

